# Two-Dimensional Clusters of Colloidal Particles Induced by Emulsion Droplet Evaporation

**DOI:** 10.3390/nano10010156

**Published:** 2020-01-16

**Authors:** Hai Pham-Van, Linh Tran-Phan-Thuy, Cuong Tran-Manh, Bich Do-Danh, Hoang Luc-Huy

**Affiliations:** Department of Physics, Hanoi National University of Education, 136 Xuanthuy, Caugiay, Hanoi 100000, Vietnam

**Keywords:** two-dimensional cluster, pickering emulsion, colloidal molecule, droplet evaporation

## Abstract

The minimization principle of the second moment of the mass distribution ($M_{2}$) is responsible for the unique structure of three-dimensional clusters by using emulsion droplet evaporation. Herein we study the structure of two-dimensional clusters of colloidal particles bound at the interface of liquid droplets in the plane. We found that, differently from the three-dimensional system, the two-dimensional clusters have multiple degenerate configurations (isomers). An interesting feature of such two-dimensional clusters is that they have the same packings as those belonging to a class of geometric figures known as polyiamonds. In particular, except for the six-particle cluster, many higher order clusters of polyiamond have not been reported previously. Using a simple geometrical approach, based on the number of ways to generate a packing, we calculated the occupation probabilities of distinct isomeric clusters. The level of agreement with the results of metropolis Monte Carlo simulations was good for clusters containing up to nine particles, suggesting that our two-dimensional cluster structures are not a result of the minimization of the second moment. In addition, the structure of these clusters is somewhat insensitive to the range and depth of the interparticle potential, in good agreement with the results in the literature.

## 1. Introduction

The formation dynamics and structures of colloidal clusters have been extensively studied because of their important role in a variety of applications, such as sensors, plasmonics and photonic crystals [1,2,3]. Furthermore, colloidal clusters that contain a small number of constituent colloids can serve as building blocks for hierarchically organized superstructures [4,5]. Manoharan et al. [6] developed an assembling route based on the use of emulsion droplets as a three-dimensional (3D) template to obtain identical colloidal clusters with high yields. In this approach, colloidal microspheres absorbed at the interface of emulsion droplets reduce the interfacial free energy. During the subsequent evaporation of the oil droplets, colloidal particles are forced to pack into clusters by capillary forces and strongly bound together by van der Waals attractions.

Following the pioneering work of Manoharan and coworkers [6], numerous studies based on emulsion droplet evaporation have been performed. Some of these focused on different types of materials/solvents [7,8,9,10], others on submicron-sized particles [11,12,13] and others still on clusters with large sizes [14,15]. Interestingly, a common feature of the small colloidal clusters is that their geometric structures are in many cases unique for a given number of constituent colloids, $n_{c}$. The uniqueness of clusters for each $n_{c}$ suggests that they are optimal packings and appear to be controlled by minimization of the second moment of the mass distribution in the cluster [16,17]. Manoharan et al. [18,19] examined the structure and free energy of colloidal clusters of hard spheres with a short-range attraction, albeit without geometrical confinement. The authors found that except in trivial cases with $n_{c}\leq 5$, the clusters possess multiple configurations whose occupation probabilities are determined by geometrical rules. In addition, rotational entropy disfavors high-symmetry clusters (octahedron occurs less frequently than poly-tetrahedron for a cluster of six particles).

Despite the strong interest in 3D cluster structures, little work has been done to investigate the emulsion-assisted formation of two-dimensional (2D) colloidal clusters. Perry and co-workers [20] studied, both experimentally and theoretically, transitions between ground states and excited states of 2D clusters of spherical colloids bound by depletion interactions. The ground state clusters adopt three distinct configurations that are dictated primarily by symmetry (permutational entropy). Because the number of ground and excited states increases rapidly with $n_{c}$, the authors considered only the case of clusters made of six particles. The experimental studies of Hurd and Schaefer [21] and Oneda [22] on colloidal suspensions, confined to two dimensions at the air–water interface, showed, depending on the strength of attractive forces, either an irreversible or reversible clustering process. However, the authors did not identify the observed cluster structures. Iwamatsu [23] analyzed the lowest energy structure of 2D colloidal clusters as a function of $n_{c}$ using a realistic model potential, which consists of a short-ranged van der Waals attractive interaction and a long range dipolar electrostatic repulsion. Although all of the predicted cluster configurations match exactly those of Lennard–Jones 2D clusters, they are not always consistent with experimental structures [24,25]; for example, for $n_{c}=7$ the predicted cluster adopts a regular hexagonal structure, whereas the experimentally observed cluster forms a circular shell structure. Note that the particles self-assemble spontaneously into such clusters, which are just intermediate states during the formation/dynamics of larger assemblies, because there is no restriction on the size of a cluster driven by attractive forces.

Polyiamonds are plane figures formed by joining congruent equilateral triangles edge to edge. Polyiamonds are considered distinct (free polyiamonds) if they are different in shape—more specifically, they are invariant under reflection, translation and rotation. The number of free polyiamonds without holes as a function of $n_{t}$, where $n_{t}$ is the number of equilateral triangles, is given by the Online Encyclopedia of Integer Sequences (OEIS): A070765 [26]. Polyiamonds, one problem in combinatorial geometry, have applications in statistical mechanics of macromolecules [27] and percolation [28]. However, to our best knowledge, there is no report about the occupation probability of these polyiamonds of a given $n_{t}$, and a connection between polyiamonds and colloidal clusters.

In this work, similar in spirit to a model originally developed for 3D systems [29,30], we formulate a model of colloid-droplet mixtures in 2D. The structures and occurrence probabilities of small clusters of colloidal particles assembled in the spherical confinement of emulsion droplets are analyzed. We find that each cluster of $n_{c}$ particles possesses a unique configuration for $n_{c}\leq 5$, but multiple, degenerate configurations for $n_{c}>5$. We suggest that the known ground state clusters for systems of particles interacting through the weak depletion interaction [20] are also relevant to colloidal clusters assisted by evaporating emulsion droplets. In particular, our cluster structures exactly correspond to those of polyiamonds.

This paper is organized as follows. In Section 2 we introduce the model and simulation methodology a binary colloid-droplet mixture. Our results are presented in Section 3. Conclusions are given in Section 4. A detailed description of polyiamonds is given in Appendix A.

## 2. Model and Simulation Method

**Figure 1:**
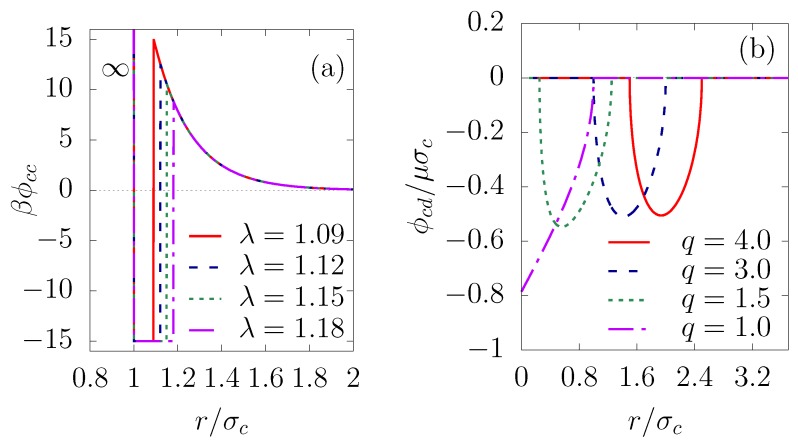
(**a**) Colloid–colloid pair potential, $\phi_{cc}\left(r\right)$ is the sum of attractive and repulsive parts (see Equation (1)), at $\beta \epsilon_{\mathrm{SW}}=15$, λ=1.09, $\beta \epsilon_{Y}=25.7$, $\kappa \sigma_{c}=5$, with $\beta =1/k_{B}T$, where $k_{B}$ is the Boltzmann constant and *T* is the temperature. (**b**) Colloid-droplet pair potential at various size ratios—$q=\sigma_{d}/\sigma_{c}=4.0$, 3.0, 1.5 and 1.0—having a minimum at the droplet interface in order to induce the Pickering effect (see Equations (2) and (3)). The value of the interfacial energy is of order $100\;k_{B}T$, so that the colloids are confined efficiently to the droplet surface. Both are scaled by the hard-sphere diameter of the colloids $\sigma_{c}$.

We consider a binary mixture of $N_{c}$ colloidal particles of diameter $\sigma_{c}$ and $N_{d}$ droplets of diameter $\sigma_{d}$ in the plane. Similar to three dimensional (3D) systems that have been studied [30], the colloids interact via a long-range screened Coulomb repulsion (Yukawa potential) and short range attraction, which can be expressed as

(1) $$\begin{array}{c} \phi_{cc}\left(r\right)=\left\{\begin{array}{cc} \infty & r<\sigma_{c}\\ -\epsilon_{\mathrm{SW}} & \sigma_{c}<r<\lambda \sigma_{c}\\ \epsilon_{Y}\frac{\exp[-\kappa \left(r-\sigma_{c}\right)]}{r} & \mathrm{otherwise}, \end{array}\right. \end{array}$$

where $\epsilon_{\mathrm{SW}}$ is the depth of the square-well potential and $\lambda =1+\Delta /\sigma_{c}$ with Δ the width of the attractive well. Two any colloids form a bond when their center-to-center separation is smaller than or equal to $\lambda \sigma_{c}$. We chose the simulation parameters to have a sufficiently large attractive component of the potential function, so that physical bonds between colloids, once formed, would be irreversible, to mimic short-range van der Waals. The parameter $\epsilon_{Y}$ measures the strength of the Yukawa repulsion and the inverse shielding length κ controls the range of the Yukawa interaction. Models of this type, as illustrated in Figure 1a, have proven useful in studies of the formation of colloidal shells [31] and the clustering of colloidal particles [29,30,32,33].

Emulsion droplets stabilized by colloidal particles, so-called Pickering emulsions, are considered to be highly stable, due to a significant reduction in the surface free energy [34]. The loss of the interfacial energy, dependent on the particle size, surface tension and equilibrium three-phase contact angle, is typically of the order of hundreds or millions of $k_{B}T$ [35]. To model this effect of the interfacial energy, Schwarz et al. [29] first introduced a colloid-droplet energy function which is related to the surface tension γ by γS, where *S* is the droplet surface that is covered by the colloid (details in [29]). In an analogous fashion, for two-dimensional systems, we consider a line tension instead of the surface tension and a contact line instead of the contact surface. Thus, the colloid-droplet energy can be expressed for $\sigma_{d}>\sigma_{c}$, as

(2) $$\begin{array}{c} \phi_{cd}\left(r\right)=\left\{\begin{array}{cc} -\mu l & \frac{\sigma_{d}-\sigma_{c}}{2}<r<\frac{\sigma_{d}+\sigma_{c}}{2}\\ 0 & \mathrm{otherwise}, \end{array}\right. \end{array}$$

and $\sigma_{d}<\sigma_{c}$,

(3) $$\begin{array}{c} \phi_{cd}\left(r\right)=\left\{\begin{array}{cc} -\mu \pi \frac{\sigma_{d}}{2} & r<\frac{\sigma_{c}-\sigma_{d}}{2}\\ -\mu l & \frac{\sigma_{c}-\sigma_{d}}{2}<r<\frac{\sigma_{c}+\sigma_{d}}{2}\\ 0 & \mathrm{otherwise}, \end{array}\right. \end{array}$$

where μ is the line tension and *l* is the length of the contact line, given by

(4) $$\begin{array}{c} l=\frac{\sigma_{d}}{2}\cos^{-1}\left[\frac{1}{\sigma_{d}r}\left(\frac{\sigma_{d}^{2}}{4}-\frac{\sigma_{c}^{2}}{4}+r^{2}\right)\right]. \end{array}$$

The interfacial energy for various droplet-colloid size ratios is pictured in Figure 1b, from which it can be seen that the potential shape is very similar to that in the three-dimensional case [29]. Moreover, for simplicity, we assume that the coalescence of the droplets is negligible. The droplet–droplet interaction has a hard-core repulsion of effective diameter $\sigma_{d}+\sigma_{c}$ to avoid one colloid being shared by two droplets.

We performed kinetic Monte Carlo (MC) simulations of colloid-droplet mixtures in the canonical ensemble. The trial state was generated by giving the particles small maximum displacements, $0.01\sigma_{c}$ for the colloidal particles and $0.01\sigma_{c}\sqrt{\sigma_{c}/\sigma_{d}}$ for the droplets. This ensured that kinetic Monte Carlo simulations approximated Brownian dynamics simulations [36]. The evaporation dynamics were introduced by forcing the droplets diameter $\sigma_{d}$ to shrink at a fixed rate. As a result, the resulting transient structures during evaporation were out-of-equilibrium. The evaporation rate was chosen so that all the droplets vanished after one-half of the simulation time ($5\times 10^{6}$ MC sweeps). This left another $5\times 10^{6}$ MC sweeps to investigate the (quasi-)stability of the clusters against thermal fluctuations. During the span of the MC simulations, a cluster, a series of colloids connected to each other by a path of bonds, can be formed spontaneously or formed via the evaporation of the emulsion droplets. Therefore, the collective modes of motion of particles in the cluster, i.e., collective translational and rotational cluster moves [37,38], are taken into account. In a MC cluster move (and its reverse), the clusters are rotated around a random rotation axis with a maximum angle $\theta_{\mathrm{cls}}^{r}=0.01\sigma_{c}/\sigma_{\mathrm{cls}}$ and translated with a maximum linear displacement $d_{\mathrm{cls}}^{t}=0.01\sigma_{c}/\sqrt[{4}]{n_{c}}$ with $n_{c}$ number of constituent colloids in the cluster and $\sigma_{\mathrm{cls}}$ the effective diameter of the cluster taken to be $\sigma_{\mathrm{cls}}=\sqrt{n_{c}}\sigma_{c}$. The choice of translational and rotational motions that satisfies the conditions for the translational diffusion constant (as discussed later) is to approximate a hydrodynamic damping of a circular cluster. Moreover, these cluster moves are proposed and accepted according to the principle of detailed balance; i.e., any cluster–cluster aggregation or cluster–particle aggregation is rejected [29].

The simulations were performed in a cubic box with periodic boundary conditions for 500 colloidal particles with packing fraction $\eta_{c}$, and at a fixed droplet packing fraction $\eta_{d}$ for a number of droplets of 6–12. For a given set of parameters, the statistical data of the sampled quantities were analyzed by running 30 independent simulations. To study the role of the interaction range on the cluster size distribution and cluster structure, we chose the width of the attractive well Δ in the range 0.09–0.21. Additionally, it is known that the cluster size distribution is driven by various factors, including (i) the droplet (colloid) packing fraction, (ii) the initial droplet size, (iii) the interaction parameter and (iv) the rotational and translational dynamics of clusters. Therefore, for a large parameter space, we restricted ourselves to simulation parameters ($\eta_{c}=0.05-0.15$, $\eta_{d}=0.15$, $\sigma_{d}\left(0\right)=8\sigma_{c}$ and the interaction parameters given in the caption of Figure 1) that provide a distribution of small-size clusters ($n_{c}\leq 9$).

## 3. Result and Discussion

Here we roughly estimate the physical time per MC sweep via the diffusion coefficient because this quantity provides a link between the mean square displacement of the particles and the simulation time. The self-diffusion coefficient of clusters $D_{\mathrm{cls}}$ is defined by the Einstein relationship [39]:

(5) $$\begin{array}{c} 2D_{\mathrm{cls}}\tau =\lim_{n\to \infty }\frac{1}{2n}\langle ▵r_{\mathrm{cls}}^{2}\left(n\right)\rangle , \end{array}$$

where *n* is the number of MC sweeps and τ is the physical time per MC sweep. The mean square displacement of the clusters after *n* sweeps, $\langle ▵r_{\mathrm{cls}}^{2}\left(n\right)\rangle$, is given by [30]

(6) $$\langle ▵r_{\mathrm{cls}}^{2}\left(n\right)\rangle =\frac{1}{N_{n_{c}}}\sum_{i=1}^{N_{n_{c}}}{\left(▵r_{\mathrm{cls},i}\left(n\right)\right)}^{2},$$

where $N_{n_{c}}$ is the number of clusters containing $n_{c}$ colloids, and $▵r_{\mathrm{cls},i}\left(n\right)$ is the displacement of a cluster from its center-of-mass after *n* sweeps.

For two-dimensional liquids, unlike three-dimensional liquids, the validity of the Stokes–Einstein relation that relates the diffusion coefficient of a Brownian particle and the fluid shear viscosity $\eta_{\mathrm{cls}}$ over a wide range of density or temperature is still unclear [40]. Hence, instead of using the two-dimensional Stokes–Einstein relation [40], $D_{\mathrm{cls}}=k_{B}T/1.69\pi \eta_{\mathrm{cls}}$, which is only valid at high densities [41], we calculate the self-diffusion coefficient from the Enskog equation in the first Sonine approximation; that is [42,43],

(7) $$\begin{array}{c} D_{\mathrm{cls}}=\frac{1}{2\rho \sigma_{\mathrm{cls}}g_{cc}\left(\sigma_{c}\right)}{\left(\frac{k_{B}T}{\pi m_{c}}\right)}^{1/2} \end{array}$$

Here $\rho =4\phi_{c}/\pi \sigma_{c}^{2}$ is the number density; $m_{c}$ is the colloid mass; and $g_{cc}\left(\sigma_{c}\right)$ is the equilibrium pair correlation function at contact. The relevant mesoscopic time scale is the Brownian time $\tau_{B}=\sigma_{\mathrm{cls}}^{2}/D_{\mathrm{cls}}$, which is the time required for an isolated cluster to diffuse over a distance equal to its diameter, where the diameter of the circular cluster $\sigma_{\mathrm{cls}}=\sqrt{n_{c}}\sigma_{c}$. An estimate of τ in terms of $\tau_{B}$ is, therefore, given by

(8) $$\frac{n\tau }{\tau_{B}}≃\frac{\langle ▵r_{\mathrm{cls}}^{2}\left(n\right)\rangle }{4n_{c}\sigma_{c}^{2}}$$

with

(9) $$\begin{array}{c} \tau_{B}=2n_{c}\rho \sigma_{c}^{3}g_{cc}\left(\sigma_{c}\right){\left(\frac{\pi m_{c}}{k_{B}T}\right)}^{1/2} \end{array}$$

At room temperature, for the clusters containing six colloidal particles and typical values of $m_{c},\sigma_{c}$, we found that $\tau_{B}\approx 10^{-2}$ s and nτ≈10 s; those values are much smaller than the realistic long-time dynamics in experiments (usually the order of minutes or hours). Despite the difference in timescale between the simulation and experiments, we do not expect the timescale to affect the final cluster structures [30].

**Figure 2:**
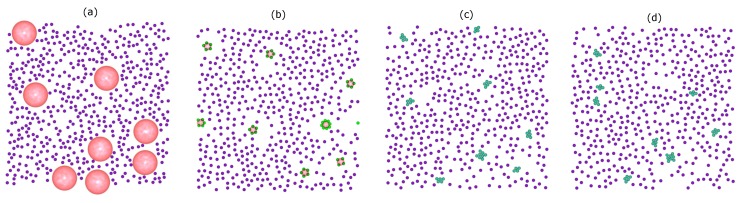
Representative snapshots from simulation trajectories: (**a**) initial state, (**b**) $3.25\times 10^{6}$ MC sweeps, (**c**) final state (after $10^{7}$ MC sweeps) and (**d**) an additional run of $10^{7}$ MC sweeps to check the (quasi-)stability of the clusters formed. Droplets are depicted as pink spheres; colloidal particles are colored according to their states during the simulation process: free colloids (purple), colloids adsorbed at the droplet surface (green), colloids in a cluster (cyan).

Figure 2 shows representative snapshots of the simulation trajectory for the binary mixture of colloids and droplets. The initial configuration consists of non-overlapping of free colloids (purple) and droplets (pink) (Figure 2a). After $3.5\times 10^{6}$ MC sweeps, accompanied by the slow evaporation of the droplet phase, a statistical number of particles is strongly confined in the droplet surface (Figure 2b). Since the droplet diameter $\sigma_{d}$ changes continuously during the simulation, the system is not at equilibrium. After this out-of-equilibrium assembly, a process towards equilibrium starts, which leads to the rearrangement of the particles to a cluster. In reality, stable clusters can be achieved only when the system is close to its equilibrium structure, a structure that forms on a very long timescale. The presence of the droplet-induced clusters as a final phase with respect to thermal fluctuations is shown in Figure 2c at the end of the simulation (after $10^{7}$ MC sweeps). As a test, we performed an additional run with $10^{7}$ MC sweeps and observed no change in the number of resulting clusters and none in their structures (Figure 2d).

Another feature is that the minimum separation between colloids at the initial stage of the simulations is larger than the bond length. In order to form a bond, the colloids must be provided with sufficient thermal energy to cross the repulsive energy barrier ($15\;k_{B}T$) of the colloid–colloid interaction. This probability is very small, and consequently, in the restricted simulation timescale, the colloidal clusters can be considered to be (quasi-)stable. However, for longer times, further clustering might occur.

The geometric structure of clusters as a function of the number of constituent particles in the plane has been studied theoretically. The optimal packings are obtained using the Lennard–Jones potential [23], minimal second moment of the mass distribution ($M_{2}=\sum_{i=1}^{n_{c}}{\left|r_{i}-r_{\mathrm{cm}}\right|}^{2}$, where $r_{i}$ is the position of the particle *i* and $r_{\mathrm{cm}}$ is the position of the cluster center-of-mass) [44,45] and depletion interaction [20].

**Figure 3:**
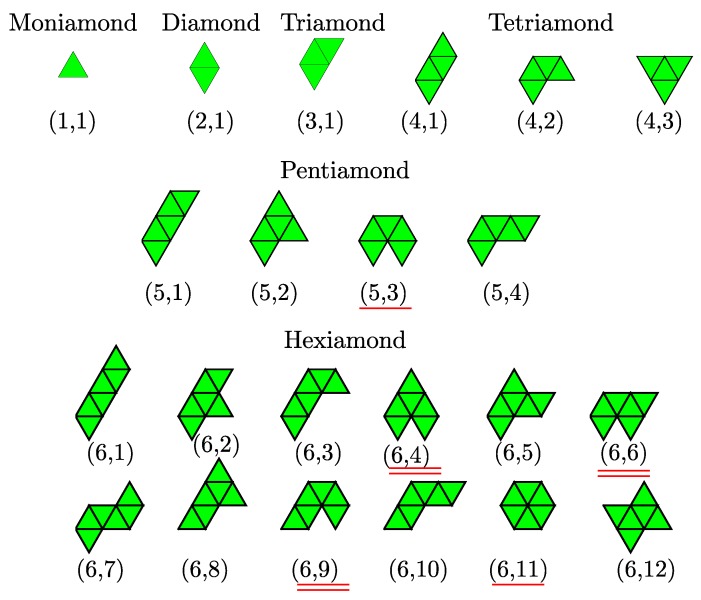
Elements of $n_{t}$-iamonds for $n_{t}\leq 6$. Each structure is labeled by the pair $\left(n_{t},m\right)$ corresponding to its number of congruent equilateral triangles and the tile number for that $n_{t}$, respectively. The graphics were produced with MATHEMATICA following [46]. Above are the names of the polyiamonds. The two (5,3) and (6,11) configurations (single-red underline) are equivalent under rotational symmetry if only their set of vertices rather than their shape is considered. Similarly, (6,4), (6,6) and (6,9) fall into a group (double-red underline).

The geometry of two-dimensional Lennard–Jones clusters where the particles interact via the Lennard-Jones potential was predicted based on genetic algorithm [23]. The first few Lennard–Jones clusters are moniamond for $n_{c}=3$, diamond for $n_{c}=4$, triamond for $n_{c}=5$, tetriamond (chevron) for $n_{c}=6$, pentiamond ($n_{c}=7$), and hexiamond (hexagon) for $n_{c}=8$. Notably, all of predicted cluster structures, which can be regarded as a spreading of triangular networks, seem to be the same as those of $M_{2}$-minimal clusters for a given value of $n_{c}$ [23,44]. The minimum colloid–colloid pair interaction, which is the sum of van der Waals attraction and dipolar repulsion, also produces configurations identical to that of both the Lennard–Jones cluster and $M_{2}$-minimal cluster [23]. However, for hard-discs interacting via a short-ranged depletion attraction, the geometric structure of clusters, for example, for $n_{c}=6$, adopts three different ground state configurations; namely, parallelogram, chevron and triangle [20]. We note that three such configurations are known as the tetriamond (4-iamond). A unique set of $n_{t}$-iamond sequences for $n_{t}$ up to 5 are illustrated in Figure 3; each case is indexed by a pair $\left(n_{t},m\right)$, where $n_{t}$ is the number of equilateral triangles and *m* is the index of the configuration in the $n_{t}$-iamond sequence. Hence, for a given $n_{t}$, the maximum value of *m* corresponds to the number of different configurations in each *n*-iamond. As shown in Figure 3, there is only one configuration for the moniamond ($n_{t}=1$), diamond ($n_{t}=2$) and triamond ($n_{t}=3$), while the tetriamond ($n_{t}=4$), pentiamond ($n_{t}=5$) and hexiamond ($n_{t}=6$) possess three, four and twelve possible configurations, respectively. The number of distinct polyiamonds of size $n_{t}$ increases rapidly as $n_{t}$ increases. Elements of the sequence for $n_{t}=7$ and 8 are given in Appendix A. It should be noted that the polyiamonds mentioned above are considered distinct if they have different number of equilateral triangles or different shapes. However, if account is to be taken of the number of vertices or point group rather than the number of equilateral triangles, then a few configurations in the sequence, e.g., the configurations (6,4), (6,6) and (6,9), are equivalent to each other. In the present work, we classify the colloidal clusters obtained via emulsion droplet evaporation by referring to the models mentioned above.

**Figure 4:**
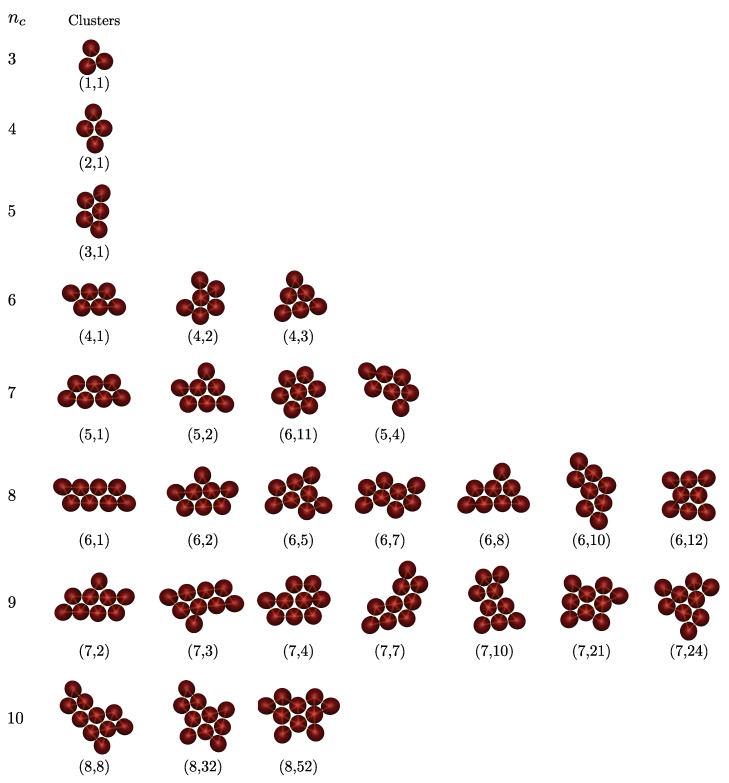
Cluster structures at each $n_{c}$ obtained from computer simulations ($\eta_{c}=0.15$, λ=1.09). Not shown is the dumbbell ($n_{c}=2$). The wire frame connecting the center of each particle to its neighbors illustrates the bond skeleton. Each structure is labeled according to the nomenclature given in Figure 3, Figure A1 and Figure A2. The observed clusters have one configuration for $n_{c}<6$, but multiple configurations for $n_{c}\geq 6$, exactly corresponding to polyiamonds.

Figure 4 shows all clusters observed in simulations ($\eta_{c}=0.15$, λ=1.09). For very small clusters, i.e., $n_{c}$ = 2–5, the structures are unique depending on $n_{c}$. These structures are identical to those of Lennard–Jones clusters, $M_{2}$-minimal clusters and clusters of attractive hard discs. This indicates that the nature of the colloid–colloid potential has little effect on the optimal structure of small clusters. However, for $n_{c}=6$, in addition to a familiar configuration of a "chevron" shape (labeled as (4,2) in Figure 3), we obtain two other isomers: one is a (4,1) parallelogram and the other a (4,3) triangle. Four distinct isomeric structures are observed together for $n_{c}=7$, as pictured in Figure 4. Surprisingly, they are exactly the same as the configuration of pentiamonds (Figure 3). Of these isomeric structures, the (5,3) configuration, an equivalent version to the (6,11) configuration with a sixfold axis of symmetry, corresponds to the theoretically predicted Lennard–Jones cluster and $M_{2}$-minimal cluster. In the eighth clusters ($n_{c}=8$), we find seven isomeric structures compared to nine possible structures of hexiamonds (only nine different configurations are counted because the (6,11) and (5,3) configuration; the (6,4), (6,6) and (6,9) are equivalent to each other under point symmetry). Two missing structures are (6,3) and (6,4), in which case (6,4) has same as the structure as the Lennard–Jones cluster. For higher order clusters ($n_{c}\geq 9$), all cluster structures obtained from computer simulations are also members of the set of polyiamonds (see Appendix A), although the number of cluster structures is much less than that enumerated in the sequence of polyiamonds. For higher order clusters ($n_{c}\geq 9$), all cluster structures obtained from computer simulations are also members of the set of polyiamonds (see Appendix A), although the number of cluster structures is much less than that enumerated in the sequence of polyiamonds. This is because the number of higher order clusters, or equivalently the number of different structure types possibly formed, directly relates to cluster size distribution, which in turn is driven by various factors (i) a droplet (colloid) packing fraction $\eta_{d}\left(\eta_{c}\right)$, (ii) relative size ratio $\sigma_{d}\left(0\right)/\sigma_{c}$.

**Figure 5:**
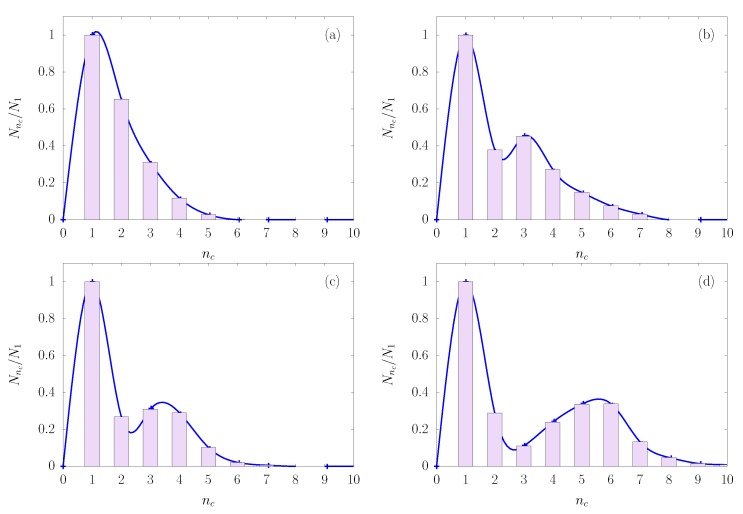
Cluster size distributions at λ=1.09 for several colloidal packing fractions (**a**) $\eta_{c}=0.05$, (**b**) $\eta_{c}=0.085$, (**c**) $\eta_{c}=0.10$ and (**d**) $\eta_{c}=0.15$. The cluster size distribution is normalized with the number of single particles $N_{1}$; the distribution is always equal to unity at $n_{c}=1$. The distributions, as shown in Figure 5a–c, therefore, displayed a monomodal characteristic at packing fractions up to 0.1, but bimodal at packing fraction of 0.15 (Figure 5d). We interpret this as a result of statistical fluctuations in a small cluster of dimers ($n_{c}=2$).

As an illustration of the role of colloid packing fraction, consider the cluster size distribution shown in Figure 5, in which $N_{n_{c}}$ is the number of clusters with $n_{c}$ colloids—normalized by dividing by the number of single particles $N_{1}$. From Figure 5a–d, the yield for high order clusters increases with increasing colloid packing fraction, signaled by a shift in cluster size distribution toward larger clusters. A similar trend can be observed in the cluster size distribution (not shown) when the interaction parameter λ decreases from 1.18 to 1.09. This result can be explained by a larger probability of capturing colloids by droplets as the colloid concentration increases. Likewise, the bonding between the constituent colloids of clusters becomes more stable against thermal fluctuations as λ decreases (see illustration in Figure 1a), leading to an increase in the yield of large clusters.

**Figure 6:**
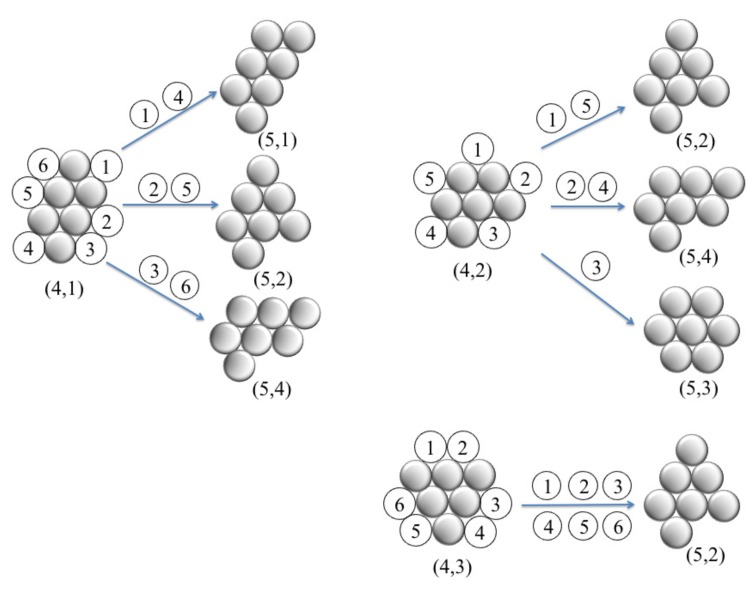
Schematic illustration of the cluster growth to calculate the occupation probabilities starting from the three six-particle clusters denoted by (4,1), (4,2) and (4,3). For the (4,1) configuration, there are six ways of inserting a particle (labeled 1–6) to construct the triangular lattice, but insertion of this particle in (1) and (4) gives two equivalent configurations (see text for details).

A previous study [20] enumerated a wide variety of cluster structures of six colloidal particles with short-ranged depletion interactions. By using complicated free energy calculations, the authors determined the occupation probabilities of ground-states and excited states, and the structural rearrangements of clusters. The cluster formation of planar triangular particles is expected to be influenced by a face-to-face contact alignment, which is quite different from a point-to-point contact alignment for spherical or disc-like particles. However, in our theoretical model, we explore the occupation probability of the clusters simply based on the number of ways to add a single particle to a given $n_{c}$-colloid cluster and with constraint that a point-to-face contact alignment is obeyed. We made this assumption based on the fact that the growth of cluster as a spreading of triangular networks was observed by the computer simulation [23] and experiments [24,25]. As an illustration, Figure 6 shows the possible configurations obtained for the three configurations (4,1), (4,2) and (4,3) by addition of one particle. In the case of (4,1), there are six ways to add one particle (labeled 1–6), but only three of them ((5,1), (5,2) and (5,4)) are inequivalent under rotational symmetries. Similarly, insertion of one particle in (4,2) can have one of three possible inequivalent configurations: (5,2), (5,3) and (5,4), whereas insertion of one particle in (4,3) produces a unique configuration; i.e., (5,2). From the number of occurrences, we calculate theoretically the probability of each inequivalent configuration (*p* (theo.)), and the results are given in Table 1. For comparison, we show the corresponding probabilities, *p* (simul.), which are directly determined from the number of times a configuration is observed through MC simulations in the final stage.

Table 1 shows that the theoretical probabilities are in good agreement with the simulation observations for configurations from (1,1) to (4,4), or, alternatively, clusters containing three to seven particles. This is intuitively reasonable, since such cluster configurations are known as ground states whose occupation probabilities are determined primarily by the rotational entropy (permutational entropy) [20]. At $n_{c}=8$, the overall agreement between the theoretical and simulated probability is generally good. However, in contrast to a high occupation probability of the (6,4) configuration predicted by the theory (20%), we have not observed the isomeric cluster of (6,4) from the present simulation. This may be explained as follows. Theoretically, there exist three types of equivalent configurations mentioned above, i.e., (6,6), (6,9) and (7,18), that contribute to the total occupation probability of the configuration (6,4). Thus, the predicted probability of (6,4) is high. However, our theoretical calculation is based on the assumption that each $n_{c}$-disc cluster containing particles is formed by addition of single particle at each triangular lattice site to clusters containing $\left(n_{c}-1\right)$ particles. Hence, the probability of finding certain cluster shape depends only on the number of ways to generate a packing. Within this model, we neglect the influence of long-ranged repulsive interaction and the presence of the droplet phase. In simulations and experiments, the repulsive interactions hinder spontaneous clustering and the droplets confine a certain number of particles at their surface. Despite a lack of consistency, our theoretical model is able to predict the simulation results quite accurately. However, it should be noted that this theory fails to provide the occurrence probability of clusters with one particle located at the center of the cluster—the internal particle, surrounded by the other particles. This is because in both experiments [6] and our simulations, while the colloids adsorbed at the droplet interface, there does not appear to be a route to an internal colloid upon collapse. A constraint subject to this route may hamper the rearrangement of particles. For $n_{c}=9$, the absence of many isomeric clusters in the simulation compared to the theoretical calculation comes from the fact that most of them have relatively low probabilities of being found, and therefore can easily be missed in the simulation data.

## 4. Conclusions

We have considered a two-dimensional suspension in which the spherical colloids and droplets are confined at the interface, making the system quasi-two-dimensional (q2D). In such a q2D system, the particle translational motion is restricted to a 2D surface and rotational motion is restricted about a line normal to the plane of the interface. We note the difference between the q2D that we studied and the q2D confinement of other studies. For example, in the work by Manahoran et al. [20], particles were confined between two coverslips with a certain spacing to generate 2D templates. Another q2D monodisperse colloid suspension was performed by construction the chamber with a thickness only slightly greater than one colloid particle diameter [47], thereby restricting the centers of the colloid particles to lie close to a plane, but allowing small amplitude deviations from that plane.

By combining the Einstein relationship and Sonine approximation, we obtaiedn a rough estimate of the simulation time scale (of order 1 s), which is typically three orders of magnitude smaller than the experimental time scale.

On the time scale over which the simulation is performed, we observed stable clusters that ranged from moniamonds to 8-iamonds. From a comparison of the six-particle ground state clusters in [20] to three possible configurations of tetriamonds, we suggest that all higher order clusters of polyiamonds ($n_{t}\leq 8$) may be expected to be ground states. In particular, using a simple geometrical rule, on the basis of permutational degeneracy, we have calculated the occupation probabilities of polyiamonds and found good agreement with those obtained from simulations. This is intuitively reasonable, since the occupation probability of ground state clusters is largely controlled by symmetry [19,20].

Unlike 3D systems, in which emulsion droplets play the role of a driving force to generate the uniqueness in cluster structures, in 2D systems droplets have little effect on stable cluster structures except that they still significantly reduce the probability of clusters that contain an internal particle. We interpret a coexistence of different structures in 2D clusters as a result of a smaller free-energy difference between those structures compared to that in 3D clusters.

Our simulation also shows that the model potentials predict the growth of clusters of a span of triangular lattices, broadly consistent with that obtained for the global minimization of the Lennard–Jones potential, the second moment of the mass distribution and an attractive van der Waals interaction. Furthermore, we found no change in the cluster structures for a variety wide of the tunable parameters of the model. These results suggest that the structures of 2D colloidal clusters are insensitive to the nature of the potential.

It is worth noting that the simulations described here could lead to the formation of uniform assembly of colloidal particles characterized by a range of well-defined sizes, shapes and structures. The availability of well-controlled assembly of colloidal particles will provide an opportunity to experimentally probe the hydrodynamic, aerodynamic and optical properties. For example, the 2D structure of polystyrene microspheres could be put to use for microlenses with controllable focal lengths [48]. In addition, some of the colloidal structures, e.g. the dimers, trimers and tetramers, can be further explored as a class of new building blocks for self-assembly to generate mesostructured systems that may find uses in practical applications, such as photonics, electronics, plasmonics and condensed matter physics [3,48].

## Figures and Tables

**Table 1:** Characteristics across multiple structure types. Each structure is represented by a symbol given in Figure 3,  Figure A1 and  Figure A2. $n_{c}$ and $n_{b}$ indicate the number of constituent particles, and the number of bonds in each structure, respectively. The point group is given in Schoenflies notation; (*p* (theo.)) is the theoretical probability, expressed as a percentage; and (*p* (simul.)) is the probability that a structure is observed via simulation.

	(1,1)	(2,1)	(3,1)	(4,1)	(4,2)	(4,3)	(5,1)	(5,2)	(5,3)	(5,4)	(6,1)	(6,2)
$n_{c}$	3	4	5	6	6	6	7	7	7	7	8	8
$n_{b}$	3	5	7	9	9	9	11	11	12	11	13	13
Point group	$D_{3}$	$D_{2}$	$C_{2}$	$C_{2}$	$D_{1}$	$D_{3}$	$C_{2}$	$C_{1}$	$D_{6}$	$C_{1}$	$C_{2}$	$C_{2}$
*p* (theo.)	100	100	100	40	40	20	13.33	49.33	8	29.33	3.81	10.13
*p* (simul.)	100	100	100	36.3	42.5	21.2	12.86	56.24	3.21	27.69	3.77	26.42
	(6,3)	(6,4)	(6,5)	(6,7)	(6,8)	(6,10)	(6,12)	(7,1)	(7,2)	(7,3)	(7,4)	(7,7)
$n_{c}$	8	8	8	8	8	8	8	9	9	9	9	9
$n_{b}$	13	14	13	13	13	13	13	15	15	15	16	15
Point group	$C_{1}$	$C_{2}$	$C_{1}$	$C_{2}$	$C_{1}$	$C_{2}$	$D_{2}$	$C_{2}$	$C_{1}$	$C_{1}$	$C_{1}$	$C_{1}$
*p* (theo.)	8.7	21.11	21.33	4.89	16.92	4.89	8.22	0.95	6.74	4.61	15.83	2.64
*p* (simul.)	0	0	26.42	9.43	11.32	3.77	22.64	0	31.58	15.79	5.26	10.53
	(7,8)	(7,10)	(7,12)	(7,13)	(7,14)	(7,15)	(7,17)	(7,19)	(7,21)	(7,22)	(7,24)	
$n_{c}$	9	9	9	9	9	9	9	9	9	9	9	
$n_{b}$	16	15	15	15	15	16	15	16	15	15	15	
Point group	$C_{2}$	$C_{1}$	$C_{1}$	$C_{1}$	$C_{1}$	$C_{2}$	$C_{1}$	$D_{2}$	$C_{1}$	$C_{2}$	$C_{3}$	
*p* (theo.)	12.32	7.21	3.02	5.29	2.2	8.45	7.37	3.71	14.83	1.24	3.56	
*p* (simul.)	0	5.26	0	0	0	0	0	0	26.32	0	5.26	

**Figure A1:**
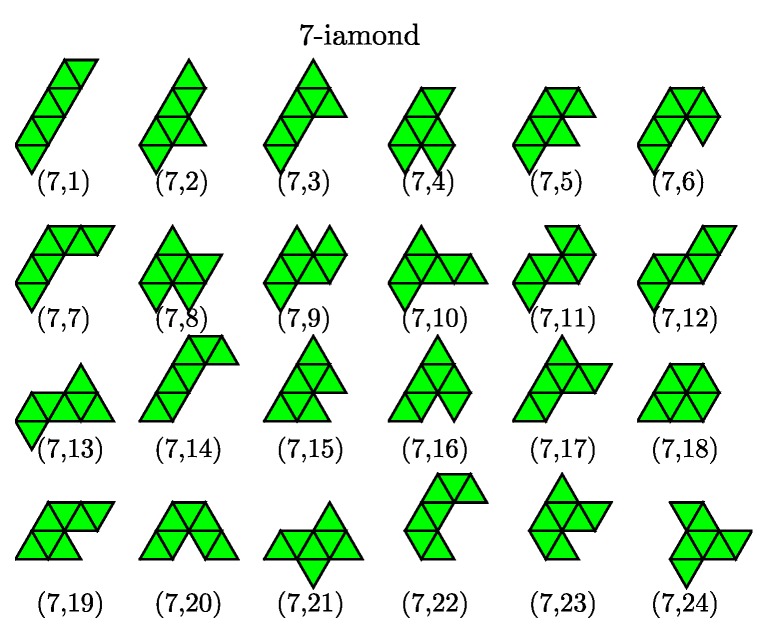
Same as Figure 3 but for $n_{c}=9$ (7-iamond).

**Figure A2:**
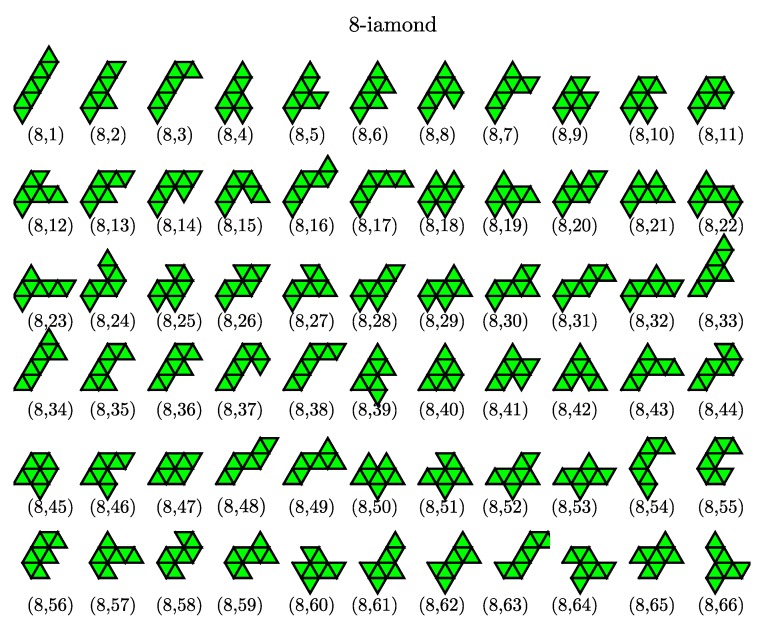
List of 8-iamonds ($n_{c}=10$).

## Appendix A

We followed the procedure of Rangel-Mondragón [46] to produce all distinct polyiamonds of a given size. A polyiamond ($n_{t}$-iamond) is a planar tile consisting of $n_{t}$ unit equilateral triangles, where each triangle is represented by a pair {A,t}; *A* is the complex number $a+b\exp(\frac{\pi i}{3})$ of the leftmost vertex of the triangle under the assumption that one of its edges rests horizontally (a,b are integers); and *t* is taken to have the value −1 or 1, such that the triangle pointing down is for −1 what pointing up is for 1. Underlying every edge-to-edge tiling of the plane by polyiamonds is a hexagonal lattice whose points can be expressed as all integral linear combinations of two numbers 1 and $\exp(\frac{\pi i}{3})$. Thus, any triangle $\left\{\left\{a,b\right\},1\right\}$ has three neighbors $\left\{\left\{a,b\right\},-1\right\}$, $\left\{\left\{a,b+1\right\},-1\right\}$ and $\left\{\left\{a-1,b+1\right\},-1\right\}$, and triangle $\left\{\left\{a,b\right\},-1\right\}$ has three neighbors $\left\{\left\{a,b\right\},1\right\}$, $\left\{\left\{a,b-1\right\},1\right\}$ and $\left\{\left\{a+1,b-1\right\},1\right\}$.

In order to generate all $n_{t}$-iamonds, we start from $\left(n_{t}-1\right)$-iamonds and proceed as follows. We take each element of $\left(n_{t}-1\right)$-iamonds and append a triangle to each of its triangles in all three possible directions. Once a full list of $n_{t}$-iamonds is obtained, we transform it into the canonical representation to remove repetitions and to sort. The two examples shown in Figure 6 and Figure A1 were drawn for $n_{t}=7$ (7-iamonds) and $n_{t}=8$ (8-iamonds), respectively.
